# On the utilization of the induced pluripotent stem cell (iPSC) model to study substance use disorders: A scoping review protocol

**DOI:** 10.1371/journal.pone.0292238

**Published:** 2023-10-12

**Authors:** Wasiri Niemis, Shenita R. Peterson, Chrisabella Javier, Amy Nguyen, Sanchi Subiah, Rohan H. C. Palmer

**Affiliations:** 1 Behavioral Genetics of Addiction Laboratory, Department of Psychology, Emory University, Atlanta, GA, United States of America; 2 Woodruff Health Sciences Center Library, Emory University, Atlanta, GA, United States of America; University of Rome La Sapienza Sapienza Faculty of Medicine and Dentistry: Universita degli Studi di Roma La Sapienza Facolta di Medicina e Odontoiatria, ITALY

## Abstract

**Introduction:**

Induced pluripotent stem cells (iPSCs) are cells derived from somatic cells via reprogramming techniques. The iPSC approach has been increasingly used in neuropsychiatric research in the last decade. Though substance use disorders (SUDs) are a commonly occurring psychiatric disorder, the application of iPSC model in addiction research has been limited. No comprehensive review has been reported. We conducted a scoping review to collate existing evidence on the iPSC technologies applied to SUD research. We aim to identify current knowledge gaps and limitations in order to advance the use of iPSCs in the SUD field.

**Methods and analysis:**

We employed a scoping review using the methodological framework first created by Arksey and O’Malley and further updated by Levac et al. and the Joanna Briggs Institute (JBI). We adopted the Preferred Reporting Items for Systematic reviews and Meta-Analyses extension for Protocols (PRISMA-P) to report items for the protocol. We searched evidence from four electronic databases: PubMed®, Embase®, Web of Science™, and Scopus®. Primary research, systematic reviews, and meta-analyses were included and limited to studies published in English, at the time from 2007 to March 2022. This is an “ongoing” scoping review. Searched studies will be independently screened, selected, and extracted by two reviewers. Disagreement will be solved by the third reviewer and discussion. Extracted data will be analyzed in descriptive and quantitative approaches, then summarized and presented in appropriate formats. Results will be reported following the Preferred Reporting Items for Systematic reviews and Meta-Analyses extension for Scoping Reviews (PRISMA-ScR) guideline and disseminated through a peer-reviewed publication and conference presentations.

**Conclusion:**

To our best knowledge, this is the first comprehensive scoping review of iPSC methods specifically applied to a broad range of addictive drugs/substances that lead to SUDs or misuse behavior.

**Registration:**

This protocol is registered on Zenodo repository (https://zenodo.org/) with doi:10.5281/zenodo.7915252.

## Introduction

Induced pluripotent stem cells (iPSCs) are a type of stem cell that are generated from adult somatic cells by genetic reprogramming technologies via transcription factors [1–3]. iPSCs possess the properties of self-renewal and pluripotency similar to embryonic stem cells (ESCs) [1, 4], enabling them to differentiate into any cell types of germ layers, i.e., ectoderm, mesoderm, and endoderm. The cellular reprogramming was firstly demonstrated by Yamanaka in mice fibroblasts using the four transcription factors of *SOX2*, *KLF4*, *POU5F1*, and *c-MYC* mediated by retrovirus in 2006 [5]. Later, he successfully reprogrammed human fibroblasts into iPSCs using the same method in 2007 [6]. Since then, there have been gradually increasing in application of reprogramming technique in various organisms and cell types, such as retinal pigment epithelium, cardiomyocytes, hepatocytes, and neurons, and become more widespread employed in the basic research and clinical application [1, 2, 7–9]. Moreover, the use of iPSCs minimizes the bioethical concerns (i.e., destruction or production of human embryos, and genetic manipulation of human embryonic stem cells [hESCs]) that are raised in hESC research [10, 11]. Nonetheless, there are ethical and legal issues for using iPSCs in research and treatments to be addressed (e.g., privacy and confidentiality of personal/genetic information, informed consent, and manufacturing and quality control of the cells) [12, 13].

The generation of iPSCs has opened a new era in biomedical health research via the comparison of the cells derived from “healthy” and “unhealthy” individuals [1, 4, 7–9]. The patient-derived iPSCs specific to a certain disease phenotype, serving as ‘disease modeling’, can unveil the biological, molecular, and developmental mechanisms of that disease and provide the future potential treatments [9, 14–19]. When combined with other technological advances such as gene editing, iPSCs have been shown to facilitate the decomposition of genetic-phenotypic correlation in order to explore the mechanisms of diseases, drug development and discovery [16, 18, 20–24].

Notably, the iPSC model has become a valuable resource for cell-based therapies and organ transplantation [9, 23, 25–27]. Advancing therapies using patient-specific iPSCs, in turn, have emerged to the translation medicine; for instance, personalized treatments [24, 27, 28] and clinical trials [29, 30]. The pioneering use of iPSCs in clinical treatment was established for retinal degenerative disease in 2014 [31, 32], and then Parkinson’s disease in 2018 [26, 33]. Later, with the progression in iPSC techniques, patient-derived iPSCs have been used in clinical trials for various diseases such as cancers [34], neuropsychiatric diseases [35, 36], regenerative diseases [25, 37] in both therapeutic and non-therapeutic purposes.

### Study rationale

#### Terms and definitions

To simplify the terms stated in this protocol, ‘substance use’ refers to any use of substances, which includes alcohol, cigarette/nicotine/tobacco, and other compounds with the potential for abuse [38]; the frequent use or abuse/misuse of a substance can sequentially lead to ‘abuse’ or ‘dependence’ (based on the Diagnostic and Statistical Manual of Mental Disorders, fourth edition (DSM-IV) [39] or International Statistical Classification of Diseases and Related Health Problems (ICD)-10/11 [40]), or ‘substance use disorder’ (SUD) (based on DSM-5 [41] diagnosis criteria). More broadly, the term ‘substance addiction’ is used to describe the most severe level of SUD diagnosis [38]. The term ‘SUD’ refers to chronic substance use that is accompanied by compulsive drug seeking and persistent use despite adverse effects [38, 42]. Throughout *this protocol*, in order to efficiently identify and map evidence that are relevant to our review topic, the term ‘SUD’ encompasses *all levels of severity across the abuse*, *dependence*, *and addiction spectrum*. See more details of inclusion criteria and search term in the **Methods**, ‘Stages I and II’ section.

#### Overview and impact of substance use disorders

Substance use disorder (SUD) or substance addiction is a common psychiatric disorder, affecting greater than 2% of the world’s population [43]. SUDs cause a high global health burden [44]. The long-term use of substances has been shown to cause adverse effects on the brain and behaviors and can lead to multiple adverse health problems as well as serious health conditions including heart and lung diseases, cirrhosis, cancers, and mental illnesses [42]. SUDs are a leading cause of death; alcohol, tobacco, and illcit drugs use disorders together account for more than 11 million lives lost annually worldwide (Global Burden of Diseases [GBD], 2019) [45]. Globally. It was estimated that 500,000 deaths (direct and indirect causes) were attributable to the use of cannabis, opioids, amphetamine, and cocaine in 2019, a 17% increase from 2009 [45]. Further, more than 70% of these deaths involved opioids and at least 30% died from opioid overdose (World Health Organization [WHO], 2023) [46].

In addition to negative health and behavior consequences, SUDs are also commonly associated with (and in some instance a cause) other illnesses including, but not limited to, spreading infectious disease (i.e., Hepatitis C and HIV/AIDS), second-hand smoke, prenatal drug exposure, automobile accidents, and unintentional injuries [42]. SUDs also have negative economic and societal consequences such as high risk of unemployment, poverty, crime, violence, and population displacement [47].

#### Current facts and trends of substance use and substance use disorders

*Declining prevalence of alcohol consumption and tobacco use*. Alcohol is the most commonly used substance worldwide. The World Health Organization (WHO) estimated 3.1 billion global population aged 15 years and older consumed alcohol in the past year and 107 million with alcohol use disorder in 2018 [48]. Alcohol consumption significantly increases risk of diseases, injuries, and mortality. The harmful use of alcohol accounts for over 2.4 million deaths in 2019 and 168,015 died from alcohol use disorder (GBD, 2019) [49]. In 2010, the WHO established the global strategy to reduce the harmful use of alcohol [50]. Since then, the global status of alcohol consumption per capita (persons aged 15 years and older) has significantly decreased from 5.7 liters per person in 2010 down to 5.5 liters per person in 2019, a 4.7% decrease from 2010 (WHO, 2023) [51].

Tobacco is the second most commonly used substance (i.e., smoke, smokeless, and electronic (e-) cigarette). Globally, in 2019, 1.14 billion people were current tobacco smokers (*excluding* smokeless and e-cigarette) and 7.69 million deaths were attributable to smoking tobacco (GBD, 2019) [52]. As a result of the WHO Framework Convention on Tobacco Control (circa 2003) [53], the rate of tobacco use steadily declined in the past 20 years. For example, the annual prevalence of current tobacco use (*excluding* e-cigarette) in people aged 15 years and older decreased from 32.7% (1367 millions) in 2000 to 22.3% (1298 millions) in 2020 (WHO, 2021) [54].

**Fig 1:**
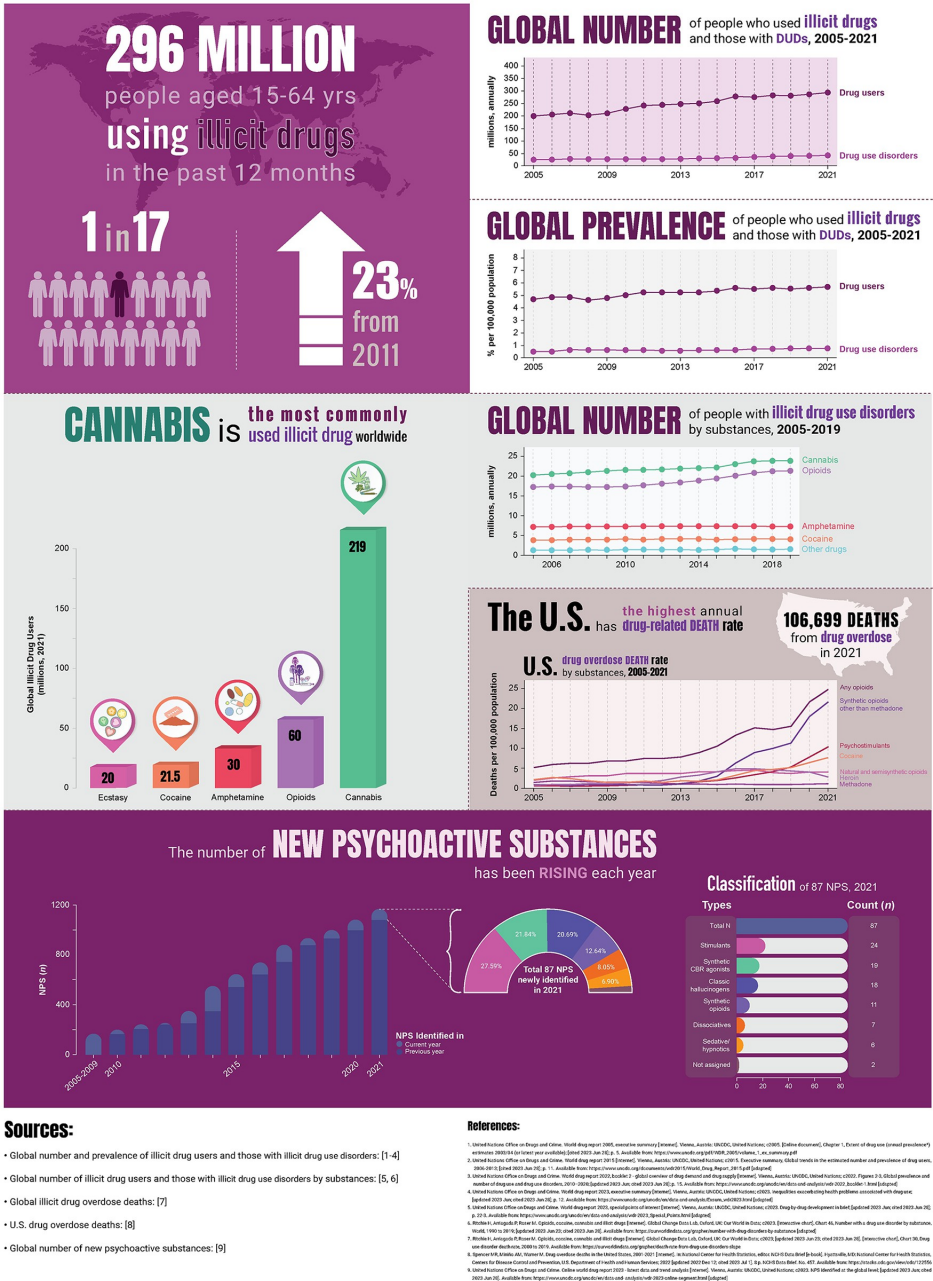
Infographic of current facts and trends on global illicit drug use.

*Rising prevalence of illicit drug use*. Based on the United Nations Office on Drugs and Crime (UNODC) report focusing on illcit drugs (*excluding* alcohol and tobacco), the prevalence of global illicit drug use continue rising (UNODC, 2023) [47]. As shown in **Fig 1** (*top* panel), in 2021, 296 million people aged 15-64 years had used an illicit drug in the past 12 months (global annual prevalence of 5.76%), 23% increasing from 240 million in 2011, whereas 39.5 million people suffered from illicit drug use disorders (DUDs) (global annual prevalence of 0.77%), 45% increasing over the past decade [47]. The most commonly illicit drug used globally is cannabis, followed by opioids and amphetamine (*middle*, *left* panel in **Fig 1**). Notably, opioids are the most harmful and fatal drug, accounting for ~70% of direct drug-related deaths [47]. The United States of America (U.S.) had the highest annual death rate from DUDs with 22.6 deaths per 100,000 population, 18% increasing from 2000 (5.3 deaths per 100,000 population) (GBD, 2019) [55] (*middle*, *bottom right* panel in **Fig 1**). The U.S. National Center for Health Statistics (U.S. NCHS) recently reported 106,699 Americans died from drug overdose (age-adjusted rate of 32.4 deaths per 100,000 population) in 2021, of which the primarily cause was related to synthetic opioids other than methadone such as fentanyl and tramadol (U.S. NCHS, 2023) [56].

This infographic presents the current facts and trends on global illicit drug use (*excluding* alcohol consumption and tobacco use). More details can be found in the **Introduction**, Study Rationale section.

**(i) Global number and prevalence of population who used illicit drugs in the past year, 2005-2021** (*top* panel). At the global level, trends in the number and prevalence of illicit drug users remain growing overtime (UNODC, 2023) [47]. In 2021, an estimated 296 million people worldwide aged 15-64 years used an illicit drug in the past 12 months (global annual prevalence of 5.76%), this was a 23% increase from 240 million in 2011, while 39.5 million people had past-year DUDs (global annual prevalence of 0.77%). These data correspond to one in 17 persons used an illicit drug and one in 100 persons suffering with DUDs in the past year [47].

**(ii) The top five commonly used illicit drugs worldwide** (*middle* panel). Cannabis is the most commonly used illicit drug globally, with 219 million users in 2021 (UNODC, 2023) [47] (middle, *left* panel); opioids were the second most commonly used drug (60 million users), followed by amphetamine (30 million users), cocaine (21.5 million users), and ecstasy (20 million users), respectively. Similar trends were observed for DUDs with the most prevalent being cannabis, followed by opioid and amphetamine, respectively (GBD, 2019) [57] (*middle*, *top right* panel). Of note, opioids account for ~70% of direct drug-related deaths [47]. The United States of America (U.S.) has the highest illicit drug-related death rates in the world (GBD, 2019) [55] (*middle*, *bottom right* panel). The age-adjusted rate of U.S. drug overdose deaths increased from 5.1 deaths per 100,000 population (14,918 deaths) in 2005 to 32.4 deaths per 100,000 population (106,699 deaths) in 2021 (U.S. NCHS, 2023) [56]. In 2021, the U.S. drug overdose death rates were significantly increased for synthetic opioids other than methadone such as fentanyl and tramadol (from 17.8 to 21.8), psychostimulants with abuse potential (from 7.5 to 10), and cocaine (from 6.0 to 7.3), whilst the death rate for heroin was decreased (from 4.1 down to 2.8) [56].

**(iii) The concern of new psychoactive substances** (*bottom* panel). New psychoactive substances (NPS), consisting of heterogenous compound of substances, cause a broad range of adverse health effects and can lead to death [58–60]. However, the prevalence of NPS use is low in the general population, but higher in adolescences and young adults, espectially in Europe [47, 58, 59, 61]. Despite the UNODC warning and monitoring, the global number of NPS is still rising every year. More than 1100 NPS have been identified since 2005, of which 87 NPS were newly identified in 2021 (UNODC, 2023) [47] (*bottom*, *left* panel). Among these 87 NPS, 85 were classified into six groups by drug effects with the most prevalent type being stimulants (27.59%), followed by synthetic cannabinoid receptor (CBR) agonists (21.84%), classic halluconogens (20.69%), synthetic opioids (12.64%), dissociatives (8.05%), and sedative/hypnotics (6.09%); the remaining two compounds yet assigned (UNODC, 2023) [62] (*bottom*, *right* panel).

DUD, illicit drug use disorder; GBD, Global Burden of Diseases; UNODC, the United Nations Office on Drugs and Crime; U.S. NCHS, the U.S. National Center for Health Statistics.

In addition, another global drug concern raised by the UNODC is ‘new psychoactive substances (NPS)’, defined as unregulated illicit substances with harm effects to health, similar effects of controlled drugs and are regulated under international drug conventions since 2014 [47, 63]. The number of NPS continues rising in the global market each year (*bottom* panel in **Fig 1**). However, NPS are still less commonly used in the general population, but the prevalence of NPS use is high in adolescence and young adults, especially in Europe [47, 58, 59, 61]. The NPS, comprising of heterogeneous group of substances, can cause broad range of adverse health effects, some of which are fatal [58–60]. There are several barriers that make it difficult to control NPS. These include (**i**) limited knowledge of NPS, especially pharmaco-kinetic and -dynamic effects, and (**ii**) under-detection or -identification of NPS drugs by routine toxicology screening. These barriers are further complicated by difficulties in monitoring demand-supply of NPS that are primarily distributed via underground or darknet marketplaces [47, 58–60].

Given the persistence of substance use and disorders globally, a multidisciplinary approach is still needed to establish proactive and permanent interaction and prevention policies within and across countries that are driven by epidemiological data. By conducting this scoping review, we hope our results will help to further identify and prioritize areas for future research that helps to reduce the global health burden for all.

#### Preliminary perspectives

*Applications of the iPSC model in the substance addiction field*. The iPSC model has been increasingly used to study psychiatric disorders [64–68], primarily schizophrenia [69–71] and bipolar disorder [72–74], but less so for SUDs during the past decade. A systematic review of iPSC technologies in psychiatric disorders [75] recently reported a small portion (10%, 6 out of total 56 papers) that focused on SUDs compared to other psychiatric disorders such as schizophrenia (23 papers) [69, 70], bipolar disorder (11 papers) [72, 73], and autistic spectrum disorder (6 papers) [76, 77]. Note that only three commonly used substances (alcohol [4 papers]; nicotine and opioid [one paper for each]) were included in McNeill et al.’s review [75]. Similarly, another line of evidence underscored the limited utilization of iPSCs to study psychiatric disorders; indeed, among substances of abuse, iPSCs have been mostly employed when studying alcohol [78].

To ensure a comprehensive survey of existing applications of the iPSC model to the substance use field, we scanned the literature using the Cochrane Database of Systematic Reviews (The Cochrane Collaboration, London, UK; https://www.cochrane.org/) and PROSPERO (University of York and the National Institute for Health Research [NIHR], York, UK). We found that there were no systematic reviews on this topic. As such, we proceeded to research the same topic in PubMed® (The National Institutes of Health [NIH], Bethesda, MD, USA), limited to studies published in English and from 1991 to 2021, using quick, gross search terms constructed by combining two strategies: “iPSC” ***AND*** “research areas of interest”. After this retrieval search (on 23 February 2022; updated on 28 December 2022), we discovered an increasing trend of cumulative evidence on iPSC and a variety of research areas, including substance use disorders (see **Supporting Information** **1A** in **S1 File**). In brief, the prevalence gradually grew after the first reprogramming of human fibroblasts launched in 2007 [6] and has been steadily rising since 2014 [32], when the first human iPSCs clinical trial occurred. However, there were fewer records of “iPSC ***AND*** substance use disorders” when compared to iPSC and other fields (see details in **Supporting Information 1B** in **S1 File**), suggesting that the iPSC model was less commonly used in SUDs. Similar to recent evidence [75, 78], these findings demonstrate that the application of the iPSC model to SUDs has been limited. Moreover, when looking into the application of the iPSC model to six commonly used substances (i.e., alcohol, amphetamines, cannabis, cigarette, cocaine, and opioid), we found that alcohol and cigarette use were the most frequently studied categories.

*Role of human-derived iPSCs in substance use research*. To further investigate how human-derived iPSCs (hiPSCs) have played a role in substance use research, we examined several records from our preliminary search of articles published from 2017 to 2022. It appears that hiPSCs were widely used in various aspects of SUD research, including disease mechanism/modeling, drug efficacy/safety/toxicity, drug response, and drug discovery. In these studies, hiPSCs were differentiated into a variety of cell types, for example, neurons (e.g., GABAergic, dopaminergic, and brain organoid), cardiomyocytes, and endothelial cells, and used in exploring different classes of substances [79–86]. In addition, the use of hiPSC model allowed researchers to examine the developmental mechanisms underlying prenatal exposure [87, 88]. Moreover, hiPSCs were leveraged in genetic and pharmacogenetic studies [86, 89–91], including personalized medicine [85, 92]. Furthermore, hiPSCs have been employed in animal-free toxicological experiments, particularly for neurotoxicity and cardiotoxicity [81, 82, 93] in addition to a conventional standard of animal models [94]. Despite these numerous implementations, the application of hiPSCs using *in vitro* cell models appears limited. One potential gap is likely due to the high investment of money and time in the generation of hiPSCs [95, 96]. Nonetheless, the data strongly shows that hiPSCs offer a promising *in vitro* cell model, which emphasizes the value of iPSC model in advancing SUD research.

#### Knowledge gap in the use of iPSC model in substance use research

Based on our preliminary search findings, we presumably conclude that there is a knowledge gap that partly arises from the lack of application of the iPSC model to studying substances of abuse. Moreover, up until now, to our knowledge, no comprehensive review of evidence on the implication of iPSC model specifically to substance use research has been reported. Given the gap, we sought to conduct a scoping review of this topic.

A scoping review is a systematic knowledge synthesis tool, which aims to map existing evidence on the topic of interests [97–100]. We opted to conduct a scoping review in order to achieve our goal of identifying knowledge gaps in the substance use field as they pertain to the application of the iPSC model. Here, we present the scoping review protocol that aims to collate the breadth of evidence, describe background knowledge on the role of iPSC model in SUDs focusing on biomedical or health science research, and ultimately identify gaps in the field.

## Methods

### Study design

This scoping review is being conducted following the methodological framework and guidelines from Arksey and O’Malley [101, 102] and the Joanna Briggs Institute (JBI) [103–108]. We will comply with the reporting guideline — the PRISMA-ScR (Preferred Reporting Items for Systematic reviews and Meta-Analyses extension for Scoping Reviews) checklist [109, 110] to report our scoping review.

### Study protocol

The study protocol is based on the methodological framework and guidelines for conduction scoping reviews [101–108]. Our protocol reported recommended items (**Supporting Information 2** in **S2 File**) in line with the Preferred Reporting Items for Systematic reviews and Meta-Analyses extension for Protocols (PRISMA-P) [107, 111, 112] for enhancing transparency and reproducibility. The draft had been reviewed and revised by all team members. The final protocol was submitted (version 1.0.0, on 9 May 2023) and publicly available on Zenodo (Genève, Switzerland; https://zenodo.org/), an open data repository, via doi:10.5281/zenodo.7915252. In addition, whenever we amend this protocol, the rational details including explanation of the update will be acknowledged and posted as an upcoming version on the protocol registry depository.

### Study framework

**Fig 2:**
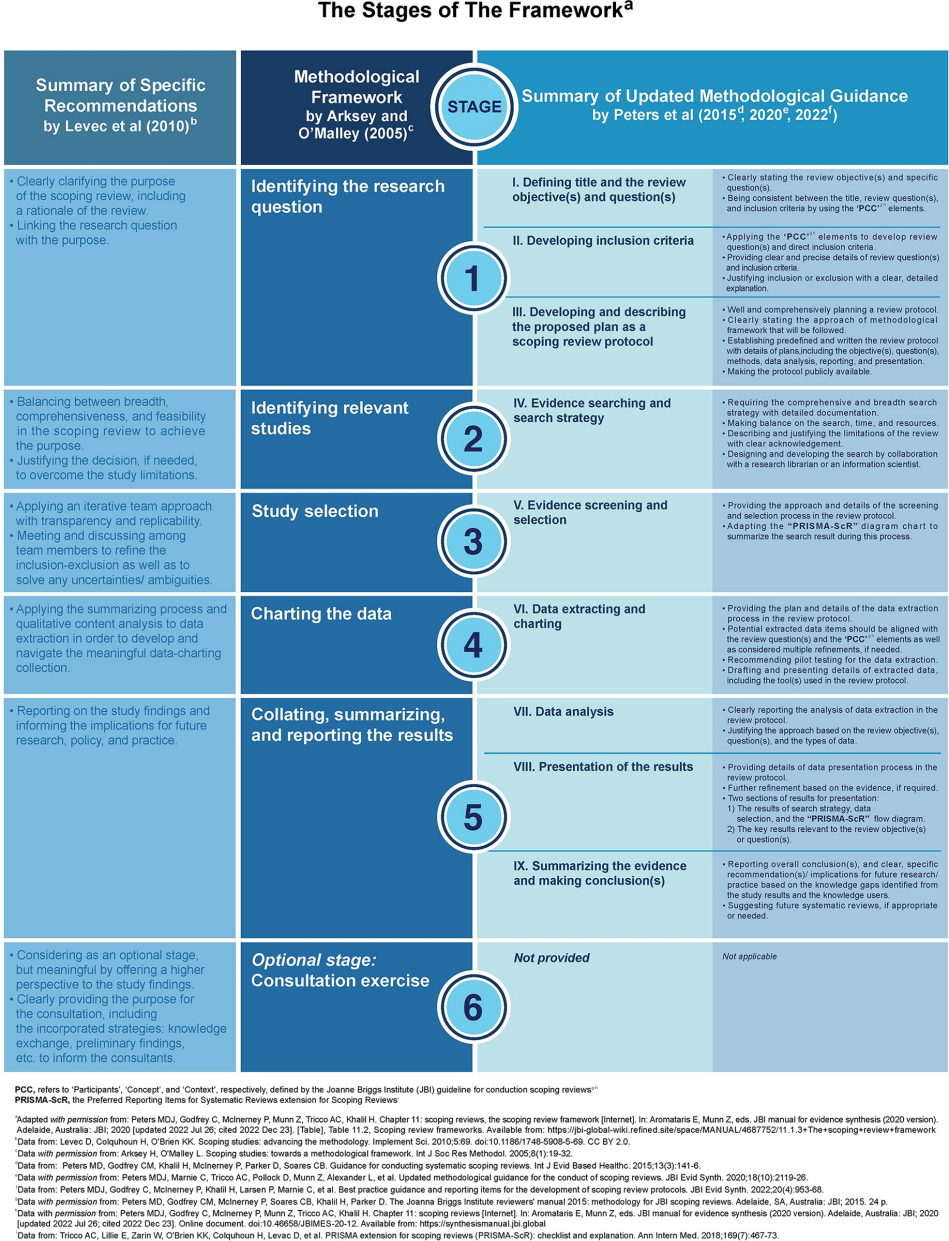
Summary of the six-stage scoping review framework.

A methodological framework of conducting scoping reviews was first proposed by Arksey and O’Malley [101], and has been periodically updated by Levac, Colquhoun, and O’Brian [102] and the Joanna Briggs Institute (JBI) [103–106] with the most recent version in 2022 [107]. Details of the framework is summarized in **Fig 2**. Briefly, our study protocol has adopted six stages of the framework [101] as follows: (**i**) **Stage 1:** identifying the research question; (**ii**) **Stage 2:** identifying relevant studies; (**iii**) **Stage 3:** study selection; (**iv**) **Stage 4:** charting the data; (**v**) **Stage 5:** collating, summarizing, and reporting the result; and (**vi**) **Optional stage:** consultation exercise. We plan to consult with experts in the areas of stem cells and substance use disorders, albeit an optional stage, stage 6 — consultation, in order to enhance the breadth of the implications of our review.

The figure shows the summary of the methodological framework to conduct scoping reviews. The scoping review framework was first introduced by Arksey and O’Malley [101], comprising of six stages as follows: **Stage 1:** identifying the research question; **Stage 2:** identifying relevant studies; **Stage 3:** study selection; **Stage 4:** charting the data; **Stage 5:** collating, summarizing, and reporting the results; and **Optional stage:** consultation exercise (see *the 2nd column*). Since then, the framework has been updated by Levac, Colquhoun, and O’Brian [102] (see *the 1st column*) and the Joanna Briggs Institute (JBI) [103–105] (see *the 3rd* and *4th columns*); the most recent version is published by the JBI group in 2022 [107]. Our review protocol has adopted all of the six stages of framework, including an optional stage 6 — consultation. Read details of each stage in the **Methods**, ‘Stages I-VI’ section.

**Stage I: Review objectives/questions. Our main objectives** are to chart and report an overview of the role of the iPSC model in substance use or addiction research including additional influencing factors (i.e., iPSC hosts, substances, and main findings) and ultimately to identify the gaps in the application of iPSC model in this field.

**Our primary question** focuses on how the iPSC model has been applied or used in substance use or addiction research?

The review will answer **the specific questions:**

**Question 1** What are detailed characteristics of the iPSC models that have been

used?

**Question 2** Which addictive drugs/substances have been studied?

**Question 3** What are the main findings/outcomes that were identified in these studies?

**Question 4** What are the gaps and limitations of the use of iPSC model in substance use or addiction research?

#### Inclusion and exclusion criteria

**Fig 3:**
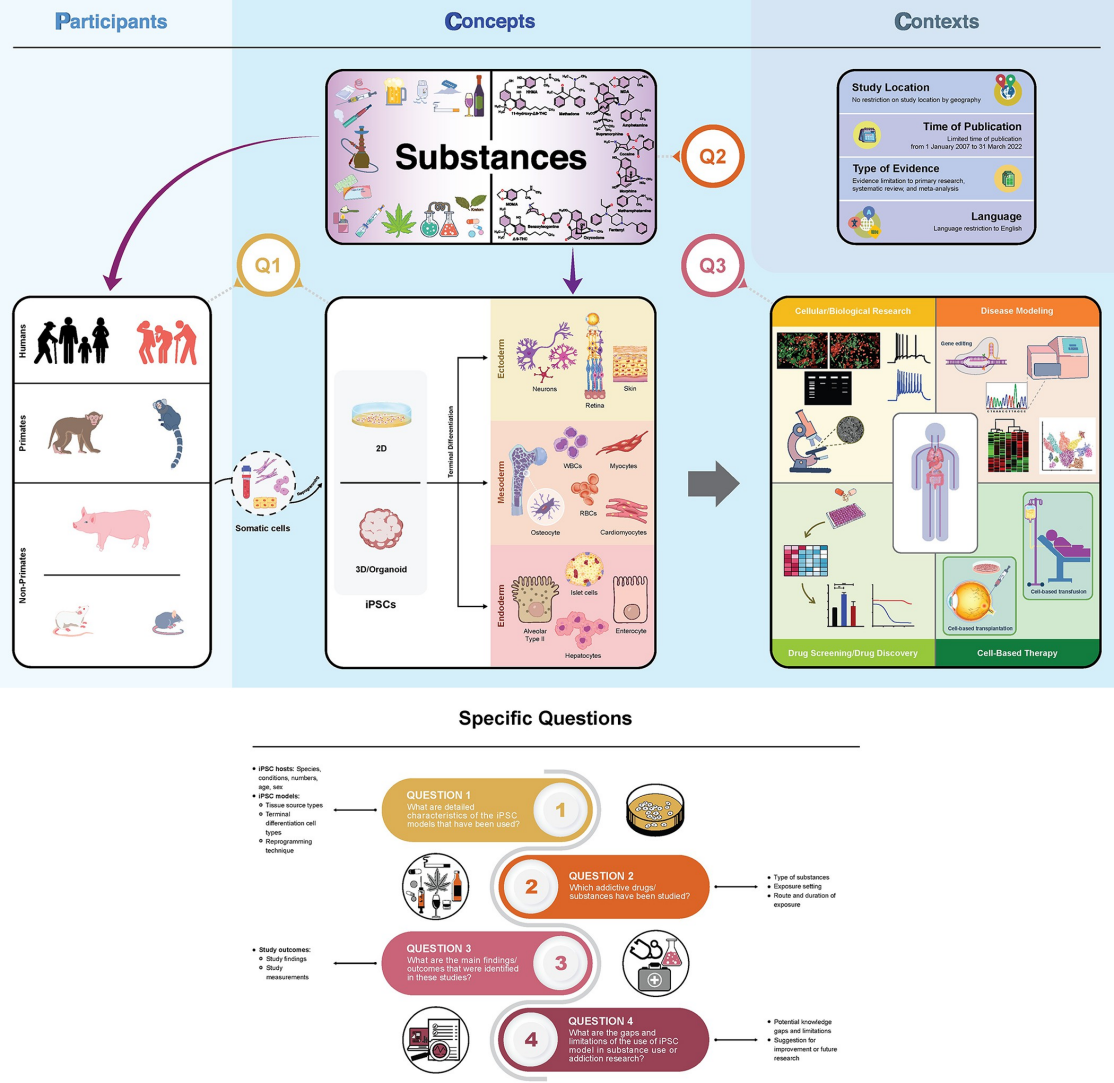
Illustration of the specific questions and PCC elements of our scoping review.

We adopted the ‘**PCC**’ — **P**articipants, **C**oncept, and **C**ontext components recommended by the JBI Manual Guidance [103, 104] on developing the inclusion and exclusion criteria. Each of the ‘**PCC**’ elements is detailed further. **Fig 3** illustrates the ‘**PCC**’ elements along with the specific questions.

The figure displays **the specific questions** along with the **PCC** elements recommended by the JBI guidance [103, 104], of which we used in order to develop inclusion and exclusion criteria for our scoping review.

The **PCC** is illustrated in a *top* panel. In brief, **P** — **P**articipants (*top left* column) refer to iPSC hosts. **C** — **C**oncepts (*top middle* column) include all of the three components: iPSC model, additive drugs or substance, and biomedical or health outcomes. **C** — **C**ontexts (*top right* column) correspond to the factors as follows: study location, time of publication, type of evidence, and language. See details of each element in the **Methods**, Stage I: ‘Inclusion and exclusion’ section.

The **four specific questions** of our scoping review are described in a *bottom* panel. **Questions 1** (**Q1**), **2** (**Q2**), and **3** (**Q3**) are presented in the **P**articipants and **C**oncepts elements (*top* panel). While, **Question 4** will provide an answer after the data extraction and data analysis have been completed. Details of the review objectives and questions including the inclusion and exclusion criteria can be found in the **Methods**, Stage I: ‘Review objectives/questions’ section.

iPSCs, induced pluripotent stem cells; RBCs, red blood cells; WBCs, white blood cells.

*Inclusion criteria*.

- **Participants:** Our review defines participants as human and non-human research subjects that were *the subjects of the iPSC model* used in the studies. Definitely, these subjects – ‘iPSC hosts’ are *donors whose tissue samples were derived into pluripotent stem cells*, which were further examined in the experiment. Non-human subjects (e.g., mice, rats, pigs, and non-human primates) are also considered. No subject restriction on gender, age, or underlying health conditions (i.e., healthy, medical conditions, SUD status, or pregnancy) are made. Disease conditions can be attributed by direct or indirect effect from the exposure of drug/substance. Note that the subjects of the iPSC model do *not* generally require a substance use/dependence diagnosis or any form of SUDs; however, the ‘iPSC hosts’ who were substance users or had SUDs seem to be of direct relevance to the review questions.
- **Concepts:** Three main concepts are required as follows:
  ○ *Intervention*: This review aims to investigate *an induced pluripotent stem cell approach*, specific to a model that induces somatic tissues to pluripotent stem cells using *the reprogramming process*. The ‘iPSC model’ can be used alone or in combination with other methodological platforms in the studies. Any iPSC-derived cell types are included.
  ○ *Drug/Substance exposure*: To address SUDs, studies must involve *addictive drugs or substances* (note that the definition of the term ‘SUD’ is descibed in the **Introduction**, Study Rationale section). The term ‘drugs/substances’ in this review are defined as *substances that cause the behavior of drug seeking and misuse* even with significantly adverse health consequence [38, 42]. Thus, in our review, the addictive drugs/substances are referred to in the following resources: (**i**) The National Institute on Drug Abuse (NIDA) [113], the National Institutes of Health (NIH), U.S. Department of Health and Human Services; (**ii**) The Substance Abuse and Mental Health Services Administration (SAMHSA) [114], U.S. Department of Health and Human Services; and (**iii**) The United States Drug Enforcement Administration (DEA) [115, 116], U.S. Department of Justice.
    In addition to well-known narcotic substances: non-controlled (i.e., alcohol and cigarettes/nicotine/tobacco) and controlled (e.g., amphetamines, cannabis, cocaine, and opioids) (according to the Controlled Substances Act (CSA) in the U.S.), other categories of drugs/substances related to addiction, dependence, or misuse (e.g., kratom and sport doping substances) including addiction treatment medications are also considered. There is no limitation on types, routes, and time (i.e., short-term and long-term) of exposure. Sources or forms (e.g., natural, synthetic, and metabolites/derivatives) of substances are not restricted. The testing condition also includes *in vitro* (laboratory testing) and/or *in vivo* (clinical/medical testing) exposure.
    It should be noted that the studies of molecular basis of substance use/addiction (e.g., nicotinic receptors, cannabinoid receptors, opioid receptors, and dopaminergic neurons) may *not* necessarily require prior drug/substance exposure.
  ○ *Outcomes*: This review searches for study outcomes or findings related or relevant to *biomedicine or health science* with an extensive scope of *direct and indirect effects from drug/substance exposure*. This includes various health issues and is not limited to specific organ or tissue involvement. The outcomes should address and provide answers to the review objectives/questions.

- **Contexts:**
  ○ No study geographic location restriction.
  ○ No limitation on study settings (i.e., academia, industries, government/federal organization, non-government organization, and combined).
  ○ Studies are in the area of biomedical research or relevant to medical or health science, for example, cellular/biological/molecular research, genetic research, drug discovery, drug response, etc. Clinical trials are also included.
  ○ Any types of study designs (i.e., observational, qualitative, and true-/quasi-experimental) are considered, however the application of *iPSC model is required* in the studies, as either a single model or a combination with other models.

Exclusion criteria.

- Studies that did not use reprogramming techniques to generate stem cells such as embryonic stem cells, without a combination with iPSC model.
- Studies that applied other experimental models such as brain slices or post-mortem brain tissues, without a combination with iPSC model.
- Studies that did not involve addictive drugs/substances or drugs/substances related to substance use behavior (i.e., addiction, dependence, or misuse).
- Studies that were not relevant to our review objectives/questions such as food and agricultures, environment, or material sciences.
- Articles that were published out of the time frame: before 1 January 2007 and after 31 March 2022.
- Articles that were not primary research, systematic review, or meta-analysis studies, i.e., book chapter, other types of review papers, protocols, guidelines, theses and dissertations, editorial or expert comments, abstract conferences, reports, and grey materials.
- Articles that were published in other languages, not in English.

#### Stage II. Evidence sources and search strategy

*Sources and type of evidence*. Our search aims to capture evidence from primary research, considered to be the best suitable resource to provide information for our review questions. We searched four electronic databases as follows: PubMed® (*The United States National Library of Medicine*, *the National Institutes of Health*, Bethesda, MD, USA), Embase® (*Elsevier*, Amsterdam, Netherlands), Web of Science™ Core Collection (*Science Citation Index Expanded*, *Social Sciences Citation Index*, *Arts & Humanities Citation Index*, *Emerging Sources Citation Index*, *Conference Proceedings Citation Index*, *Book Citation Index*, *and Current Chemical Reactions*, *Index Chemicus; Clarivate*™, London, UK), and Scopus® (*Elsevier*, Amsterdam, Netherlands) with the limited time period from 1 January 2007 to 31 March 2022. The rationale for starting our search in 2007 was because the iPSC model was first established by Yamanaka in 2006 [5]. The search was also restricted to articles published in English, since English is the only language that all team members can read.

#### Search strategy and terms

*Initial search and search development*. An initial search, led by the team leader (WN) and facilitated by team members (SRP and RHCP), was comprised of **two concepts** [search terms]: (**i**) **iPSC:** ["induced pluripotent" OR "iPS" OR "iPSC"] ***AND*** (**ii**) **addictive drugs or substances:** ["substance*" OR "addict*" OR "abuse" OR "dependence" OR "use disorder*" OR "substance use" OR "drug use"] *OR* ["alcohol" OR "cocaine" OR "opioid" OR "smoking*" OR "amphetamine*" OR "methamphetamine*" OR "marijuana" OR "cannabis" OR "nicotine" OR "cigarette"]. Our primary search was executed in the four databases: PubMed, Embase, Web of Science (WOS), and Scopus, in February 2022 (on 28 February 2022), in order to define the feasibility of existing articles relevant to our topic of interest.

The search produced a decent number (*N* = 3953) of records across the databases even when limited to the English language: PubMed (*n* = 678), Embase (*n* = 933), WOS (*n* = 935), and Scopus (*n* = 1407). The initial search terms were reviewed and refined to generate a final search strategy in order to ensure that it would identify the articles that were relevant to the objectives and questions of our scoping review.

**Fig 4:**
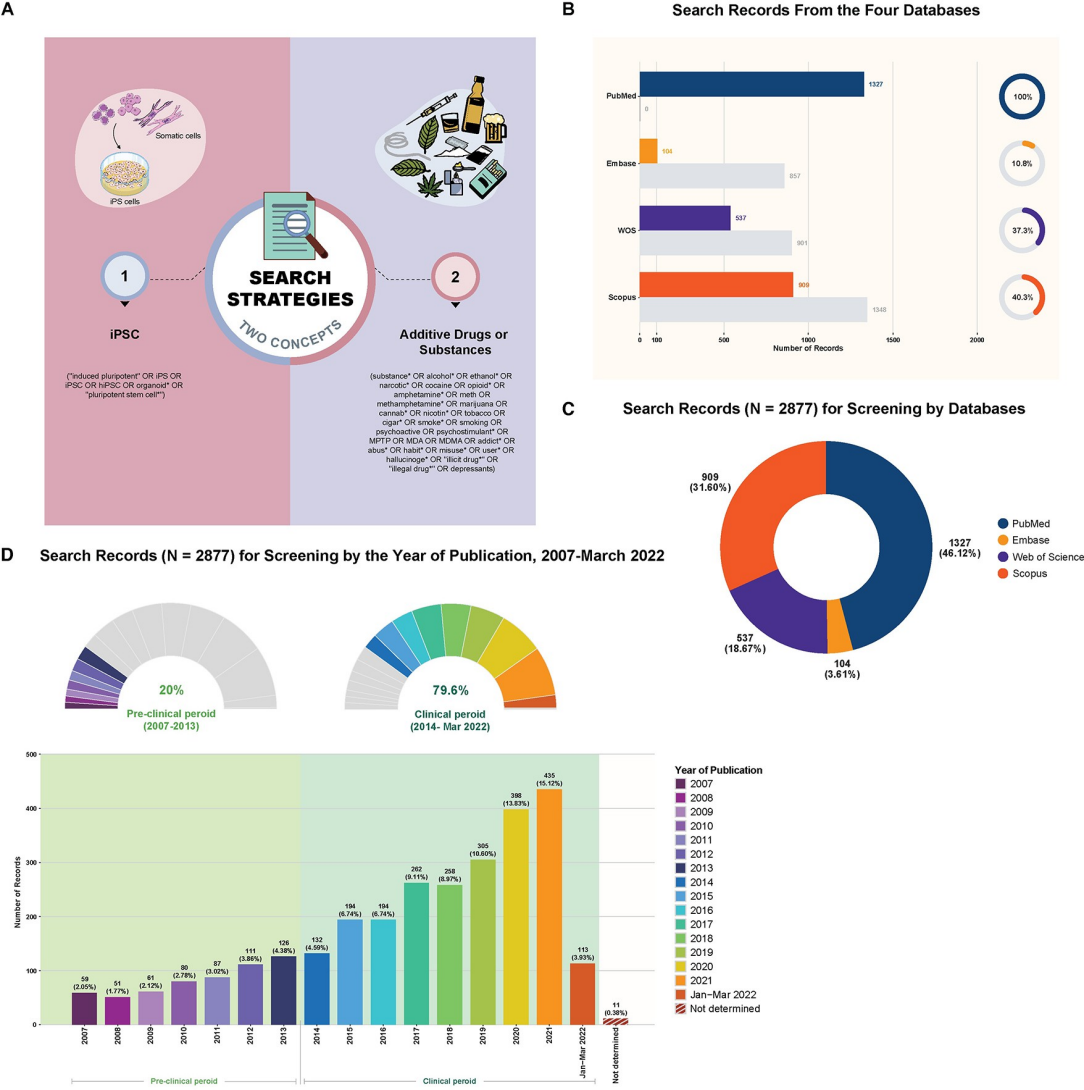
Search strategy, search terms, and summary of search records from the four electronic databases and for screening.

*Search strategy*. We applied **two concepts** with the search terms (**4A** in **Fig 4**) in the final search strategy as follows:

- **Concept 1 – iPSC:** ("induced pluripotent" OR iPS OR iPSC OR hiPSC OR organoid* OR "pluripotent stem cell*").

AND

- **Concept 2 – Addictive drugs or substances:** (substance* OR alcohol* OR ethanol* OR narcotic* OR cocaine OR opioid* OR amphetamine* OR meth OR methamphetamine* OR marijuana OR cannab* OR nicotin* OR tobacco OR cigar* OR smoke* OR smoking OR psychoactive OR psychostimulant* OR MPTP OR MDA OR MDMA OR addict* OR abus* OR habit* OR misuse* OR user* OR hallucinoge* OR "illicit drug*" OR "illegal drug*" OR depressants).

### (A) Search strategy and terms

Search strategy used in the search was comprised of **two concepts**: (**i**) **Concept 1** (*left* panel), ‘iPSC’ ***AND*** (**ii**) **Concept 2** (*right* panel), ‘additive drugs or substances’. Search terms for each concept are presented in the figure. More details including search queries for each of the four databases can be seen in the **Methods**, Stage II: ‘Search strategy and terms’ section and **Supporting Information 3** in **S3 File**.

### (B) Search records retrieved from the four electronic databases

Both the horizontal bar plot and pie charts show the distribution of records identified from the search of our scoping review, categorized by the source of electronic databases. Our search was done in **four electronic databases: PubMed**®, **Embase**®, **Web of Science**™ (WOS), and **Scopus**®. After removing duplication across the databases, the record of each database, as shown in the horizontal bar plot and pie charts, is divided into **two subsets**: **included** and **excluded** records. Colors on each subplot indicate included (also corresponding to the source of databases) and excluded records (grey).

Horizontal bar plot (*left* panel) represents the distribution of search records for each database. Each subplot shows a plot of the number of search records (x-axis) against the source of databases (y-axis).

Pie charts (*right* panel) represent the distribution of included and excluded records for each database. The numbers and percentages in each subplot indicate a portion of included records of each database.

### (C) Search records for screening by databases

The pie chart represents the distribution of the search records for screening, categorized by the source of electronic databases. Following the search, we removed duplication across **the four databases: PubMed**®, **Embase**®, **Web of Science**™ (WOS), and **Scopus**®; as a result, a total of 2877 (100%) records were advance into the screening process. Each pie slice shows a proportion of the records (presented as both numbers and percentages) for screening from each database. Colors on pie chart indicate the source of databases.

For **Fig 4B** and **4C**, the source of electronic databases, also representing included records (**4B**), is indicated by colors: PubMed®, dark blue; Embase®, light orange; Web of Science™ (WOS), purple; Scopus®, dark orange.

### (D) Search records for screening by the year of publication, 2007-March 2022

Both the bar plot and half pie chart show the distribution of search records of our scoping review for screening, categorized by the year of publication. As a search result, a total of 2877 (100%) records were moving into the screening step. Our search was limited by **the year of publication from 2007 to March 2022**, as the first reprogramming technique was generated in 2006 [5]. Moreover, according to the first clinical trial of iPSC-based therapy in retinal degenerative diseases in 2014 [31, 32], so we categorized records into **two basis periods: preclinical** (2007-2013) and **clinical** (2014-March 2022). Overall, numbers of search records per year have been significantly increasing since 2014 when the first iPSC-based clinical trials launched. Nearly 80% of total identified records were published in a clinical period (2014-March 2022). Markedly, more than twice the number of annual records (*n* > 300) were published in 2019, 2020, and 2021 than published in 2013 (*n* = 126) alone.

Half pie chart (*top* panel) represents the distribution (percentage) of records in preclinical (*left*) and clinical (*right*) periods.

Bar plot (*bottom* panel) represents total search records for screening, categorized by the year of publications. The number and percentage of records are displayed on the top of each bar. The x-axis indicates the year of publications. The y-axis indicates the number of search records. Background colors on the plot indicated preclinical (2007-2013, light green; *left* side) and clinical (2014-March 2022, green; *right* side) periods.

Colors on each bar and each section of half pie indicate the year of publication.

iPSC, induced pluripotent stem cell.

The search terms and indexed terms were adapted according to the format of each database. Full search strategies for all four databases can be seen at **Supporting Information 3** in **S3 File**. The search aimed to capture published articles of primary research, systematic reviews, or meta-analyses, which are likely to provide key evidence to answer our review questions. The final search was completed across PubMed, Embase, WOS, and Scopus by a team member (SRP), a research informationist.

Our final search identified a total of 5983 primary research records via the four electronic databases (PubMed, Embase, WOS, and Scopus) (**4B** in **Fig 4**), which were imported into EndNote™ version 20 (Clarivate™, London, UK). After removing duplicates (*n* = 3106), 2877 records remained for further study selection (**4C**, **4D** in **Fig 4**).

#### Stage III. Study selection

*Pilot testing*. We performed pilot testing prior to study selection. Inclusion and exclusion criteria were created and used for the ‘*two-step*’ approach of study selection — ‘title and abstract screening’ and ‘full-text review’. In pilot tests, each article required agreement from at least three independent reviewers to be included. When a disagreement occurred, it was resolved by a fourth reviewer and follow-up discussion. This step helps reviewers to reduce uncertainties or ambiguity as well as to refine the inclusion-exclusion criteria if needed.

For instance, prior to the beginning of the actual screening, pilot tests were conducted among four raters (WN, CJ, AN, and SS) were achieved on the same 175 random articles in total to ensure that titles and abstracts were retrieved in accordance with the inclusion/exclusion criteria. All disagreements were solved by the aforementioned options.

Furthermore, the agreement among reviewers in the process of article screening and selection has been measured using the interrater reliability (IRR). Interrater agreement is managed as follows: if the IRR value is greater than or equal to 0.80, which indicates a high or strong level of agreement [117, 118], then those articles are advanced to the next stage of the study framework. In cases where the IRR value is less than 0.80, which indicates low or poor level of agreement [117, 118], then the reconciliation of refining inclusion-exclusion criteria and pilot tests would come into play; the refining criteria and testing would be repeatedly taken until the level of agreement reaches the IRR threshold of 0.80. We have applied our interrater agreement protocol into the pilot testing, the study selection (title-abstract screening and full-text review) stage including an upcoming stage of data extraction.

#### Screening and selection

In total, 2877 search records were imported into Covidence (Melbourne, Victoria, Australia; https://www.covidence.org/), a web-based collaboration software platform for study selection. The selection is a required component of the ‘*two*‐*step*’ process, beginning by examining ‘titles and abstracts’ (*primary* screening) and subsequentially ‘full-text articles’ (*secondary* screening). After the full-text review is completed, a final set of the selected articles will be eligible for data extraction. The reasons for excluding an article during the selection process will be indicated and reported. Note that during the actual screening and selection processes, each article requires agreement from at least two independent reviewers in order to be selected. Discrepancies are resolved by the decision of a third reviewer and subsequent analysis of the reasons for disagreement among all reviewers.

**Fig 5:**
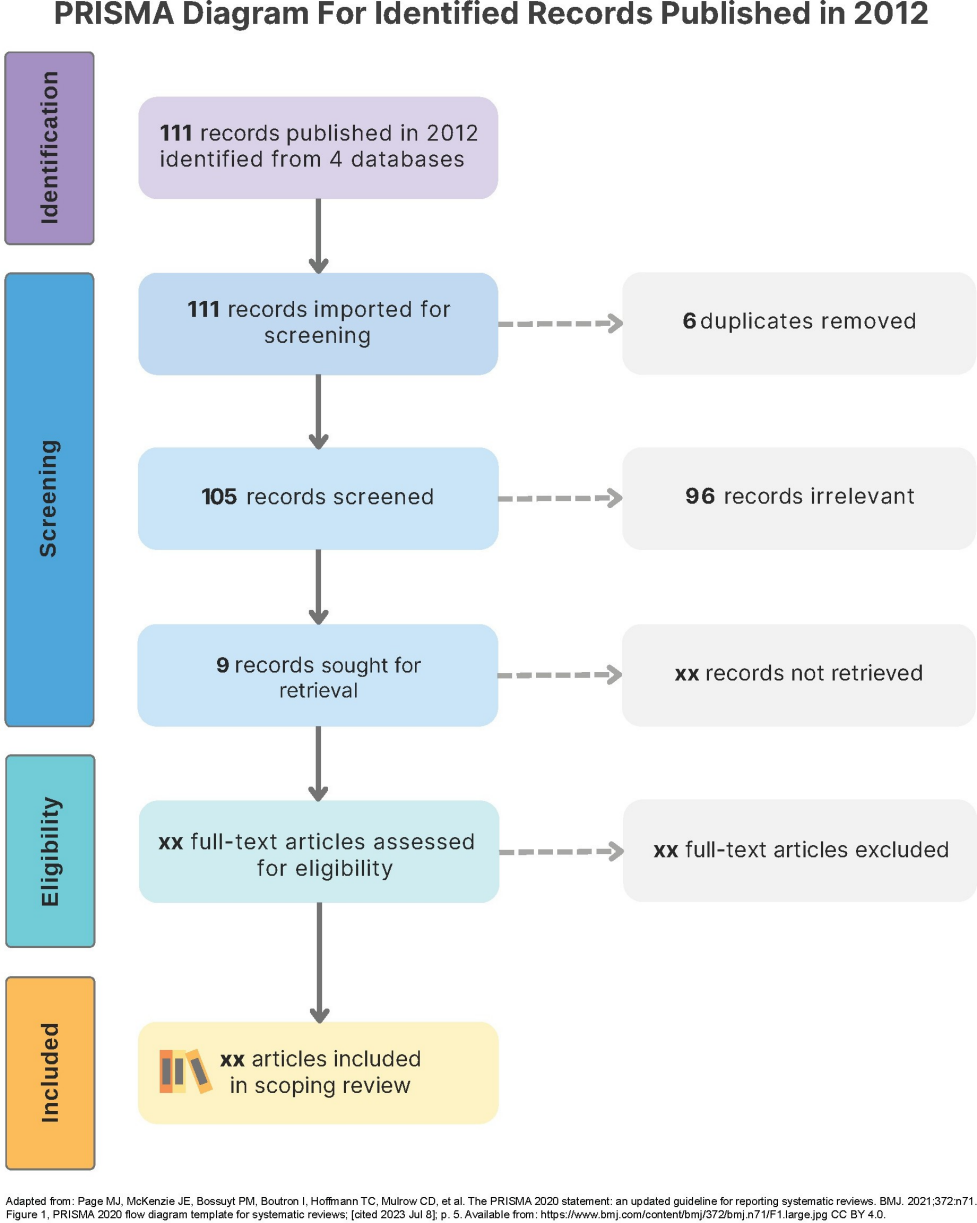
Example of a PRISMA flow diagram for the study selection of 111 records published in 2012.

Overall, records at each stage of the study selection starting from searching, title-abstract screening and full-text review, until final selection for data extraction will be summarized and presented in a PRISMA flowchart [109, 119]. This review has been *ongoing*. At the time when the protocol was prepared, the review was in the title and abstract screening process. **Fig 5** illustrates an example of a PRISMA flow diagram showing a search result of 111 (out of 2877) records published in 2012 that were completely title-abstract screened (by WN, CJ, AN, and SS), resulting 9 articles for full-text retrieval and further review.

The figure shows an example of a PRISMA flowchart [109, 119], following the guideline from the PRISMA-ScR (Preferred Reporting Items for Systematic reviews and Meta-Analyses extension for Scoping) checklist [109], for reporting the study selection of the scoping review. The diagram displays the following steps of **study selection:** study **identification**, ‘*two-step*’ **screening** with ‘*title-abstract*’ and ‘*full-text*’, determining **eligibility**, and **inclusion**. Note that this is *an ongoing review*; at the moment, we have not finished screening the entire 2877 records yet. Thus, we used the search records published in 2012 as an example. Definitely, we will present a PRISMA flow diagram [119] for our entire records, once we complete the study selection.

Here is a brief description of an example of a PRISMA flowchart. Our search result via the four databases (PubMed®, Embase®, Web of Science™ [WOS], and Scopus®) collected total 2877 records after de-duplication. Out of the 2877, 111 records were published in 2012 [**Identification**, purple] and moved further into a screening process. The 111 records were completely title-abstract screened (by WN, CJ, AN, and SS), resulting 9 records for full-text retrieval [**Screening**, blue], and then review in order to further determine eligibility [**Eligibility**, green] for inclusion [**Inclusion**, yellow] in the final review.

Fig 5 is adapted from Page et al, Fig 1, PRISMA 2020 flow diagram template for systematic reviews CC by 4.0 [119].

#### Stage IV. Data extraction

Data extraction will be conducted on selected articles after full-text review by utilizing the ‘**PICO**’ — **P**opulation, **I**ntervention, **C**omparison groups, **O**utcomes model [120, 121]. A data charting form will be developed under an iterative process in order to capture variables relevant to our review questions, and is expected to be refined, if needed, during the data extraction process [105, 108]. Extracted data will be collected using the data collection tools such as Covidence (https://www.covidence.org/) and Google Forms (Google LLC, Mountain View, CA, USA). These data will be recorded in a tubular format with textual description using the spreadsheet software such as Microsoft Excel (Microsoft Corporation, Redmond, WA, USA) and Google Sheets (Google LLC).

Each paper requires at least two reviewers independently charting data items to ensure the accuracy and consistency of all required/relevant information. Pilot tests of data extraction will be employed among all reviewers on at least the same 30 randomly eligible full-text articles. Extracted data will be compared across raters. The agreement of data extraction will be assessed throughout the data extraction process with an interrater agreement (IRR) threshold of greater than or equal to 0.80. Any controversies or disagreements will be solved by a third reviewer and discussion.

We will extract and record data in table form. Extracted data will define details as follows: (**i**) ***basic* study** information (authors, year of publication, and locations/countries where studies were conducted), and (**ii**) ***specific* study** information (study populations, aims, designs and experimental details, main outcomes, and the findings related or relevant to our review questions). Among all eligible articles, we prioritize a sub-group of those that specifically used iPSCs derived from humans; those will be extracted exclusive data as acquainting information of the implication of human iPSC model. The table outline and contents will be developed and justified based on the information obtained throughout the data extraction process. These materials will be used in next step of the data analysis. A draft of data charting form is displayed in **Table 1**.

**Table 1:** A draft of data extraction form.

**General Information**		
	Variables	Description
***Article information***	**Authors’ names**	Name of authors
	**Authors’ affiliations and locations**	Institutes/countries where authors were affiliated with
	**Year of publication**	Year of the article published
	**Article title**	Title of the article
	**Journal name**	Journal that the article published
	**Scope(s) of journal**	Scopes of a journal that the article was published
	**Article type**	**Type of the article published:** primary research, systematic review, or meta-analysis
	**Language**	Language of the article published
***Study information***	**Study site(s)**	The place(s) (countries) where the study was conducted and/or where the sample recruitment took place
	**Study designs**	• Observational study • Experimental study
	**Study setting**	• Basic research • Clinical research
	**Study aim(s)**	Aim of the study
	**Study method(s)**	**Methods used in the study:** • *iPSC model*: alone/combined with other models • *Additional models* (if applicable)
	**Study samples: primary and secondary (if applicable)**	**Samples of the study:** • *Primary samples*: iPSC hosts • *Secondary samples* (*including species*, *number*, *condition*, *age*, *and sex*, *if applicable*): samples used in other models, in addition to iPSC method
	**Main study finding(s)**	Each main finding of the study, of which not all *may* or *may not* be relevant to our review questions
	**Main finding measurement(s)**	Each main finding assessment and unit (if applicable)
	**Study conclusions**	Conclusions of the study, which *may* or *may not* be relevant to our review questions
	**Study challenges/caveats**	Challenges/caveats of the study, which *may* or *may not* be relevant to our review questions
**Specific Information**		
	Variables	Description
***Primary sample information*** **(Participants)** **Details are specific to** **primary samples, defined as iPSC hosts**	**Sample type**	**Species of iPSC hosts:** • Humans • Primates • Non-primate mammals: mice, pigs • Non-mammals: C. elegans, zebra fish
	**Sample size**	**Number of iPSC hosts**
	**Sample health condition**	**iPSC hosts’ health conditions:** • Healthy • Specific underlying diseases or health/medical conditions (if applicable): for example, chronic lung diseases, cirrhosis, and cancers, substance use disorders, etc.
	**Age (if applicable)**	**iPSC hosts’ age (mean):** days/months/years
	**Sex (if applicable)**	**iPSC hosts’ sex:** male/female
	**Ethnicity (if applicable)**	**iPSC hosts’ ethnicity:** African, Asian, European, etc.
***iPSC model information*** **(Concept - #1)** **Details are specific to iPSC model used in the study**	**Structure of iPSCs**	• 2D • 3D (organoid)
	**Original tissue type**	**Type of tissues obtained from iPSC hosts:** for example, lymphoblasts, fibroblasts, epithelial cells, etc.
	**Terminal differentiation tissue type**	**Type of derived tissues after reprogramming stage:** for example, alveolar cells, hepatocytes, neurons, etc.
	**Reprogramming technique**	**Type of reprogramming transfer system**^a^ • Integrative transfer system: viral, non-viral • Non-integrative transfer system: viral, non-viral
***Substance information*** **(Concept - #2)** **Details are specific to additive substances that used in the study**	**Type of substances**	**Type of substances** [defined following the resources^b^–^d^]: • Alcohol/ethanol • Amphetamine/methamphetamine • Cannabis/marijuana/cannabinoids • Cigarette/nicotine/tobacco • Cocaine • Opioids/heroin • Synthetic opioids: methadone, naloxone • Psychostimulant drugs: methylphenidate (Adderall®, Ritalin®) • Hallucinogens: LSD, PCP • Depressants: benzodiazepines • Tranquilizers: ketamine • Other illicit drugs: ecstasy (MDMA), MDA, MPTP, kratom, etc.
	**Categories of substances**	• **Alcohol**, **tobacco** (i.e., cigarette, e-cigarette, and nicotine) • **Controlled substances** (defined as an illegal or prescription drug regulated by the Controlled Substances Act (CSA) in the U.S.) • Narcotic/Non-narcotic • Prescribed/Non-prescribed
	**Exposure setting**	• *In vitro* • *In vivo*
	**Duration of exposure (depend on the exposure setting)**	• Short-term/long-term (if applicable) • Days/months/years (if applicable)
	**Route of exposure**	• ***Direct* exposure:** *in vitro* exposure, drinking, ingestion, injection, smoking, etc. • ***Indirect* exposure:** prenatal exposure
***Outcome information* (Concept - #3)** **Details are specific to the study outcomes relevant to the review questions** **(i.e., substances-related health outcomes)**	**Relevant outcomes**	**Results/findings relevant to the review questions:**• *Direct relevance*: direct effect of substance exposure • *Indirect relevance*: indirect effect of substance exposure such as prenatal exposure
	**Relevant outcome measurement**	Assessment of results/findings relevant to the review questions
***Specific challenges/*** ***caveats*** **Details are specific to the review questions**	**Relevant challenges/caveats**	This challenges/caveats specific or related to any of our review questions or ‘**PCC**’^e^ ^f^ elements

iPSCs, induced pluripotent stem cells

PCC, refers to ‘Participants’, ‘Concept’, and ‘Context’, respectively, defined by the Joanne Briggs Institute (JBI) guideline for conduction scoping reviews^e f^

^a^ Al Abbar A, Ngai SC, Nograles N, Alhaji SY, Abdullah S. Induced pluripotent stem cells: reprogramming platforms and applications in cell replacement therapy. Biores Open Access. 2020;9(1):121-36.

^b^ National Institute on Drug Abuse. Commonly used drugs charts [Internet]. Research topics. Rockville, MD: NIDA, National Institutes of Health; c2022 [updated 2020 Aug 20; cited 2022 Mar 4]. [about 4 screens]. Available from: https://nida.nih.gov/research-topics/commonly-used-drugs-charts

^c^ Substance Abuse and Mental Health Services Administration. Key substance use and mental health indicators in the United States: results from the 2021 National Survey on Drug Use and Health (HHS Publication No. PEP22-07-01-005, NSDUH Series H-57) [Internet]. Rockville, MD: Center for Behavioral Health Statistics and Quality, SAMHSA; 2022 [updated Dec 2022; cited 2023 Feb 5]. [about 100 screens]. Available from: https://www.samhsa.gov/data/report/2021-nsduh-annual-national-report

^d^ United States Drug Enforcement Administration. Controlled substances - alphabetical order [Internet]. Drug information, The Controlled Substances Act. Springfield, VA: United States DEA, U.S. Department of Justice; c2023 [updated 2023 Jul 7; cited 2023 Jul 12]. Online document; [20 pages]. Available from: https://www.deadiversion.usdoj.gov/schedules/orangebook/c_cs_alpha.pdf

^e^ Peters MD, Godfrey CM, McInerney P, Soares CB, Khalil H, Parker D. The Joanna Briggs Institute reviewers’ manual 2015: methodology for JBI scoping reviews. Adelaide, SA, Australia: JBI 2015. 24 p.

^f^ Peters MDJ, Godfrey C, McInerney P, Munn Z, Tricco AC, Khalil H. Chapter 11: scoping reviews [Internet]. In: Aromataris E, Munn Z, eds. JBI manual for evidence synthesis (2020 version). Adelaide, Australia: JBI; 2020 [updated 2022 Jul 26; cited 2022 Dec 23]. Online document. Available from: https://synthesismanual.jbi.global doi:10.46658/JBIMES-20-12

#### Stage V. Data analysis and presentation

The plan for data analysis and presentation will be developed and adjusted upon the data contents until the data collection is completed [106, 108]. Following data charting, we will mainly use qualitative analysis to describe and transcribe the summary of the data (see a draft of data items in **Table 1**) extracted from all included articles. Results will be reported using narrative and descriptive approaches that align with our review objectives and questions. Quantitative data analysis will be used to mainly summarize the results in the form of number and percentage. Transcribed texts, tables, charts, and figures will be presented when appropriate. Sub-group presentation will be considered and summarized according to the review questions. To keep the standard of scoping review, we will follow the PRISMA-ScR [109, 110] guideline to report our results.

Our data presentation aims to provide the summarizing evidence-based information in response to the specific review questions, and then to allow reviewers to identify the knowledge gaps as well as formulate the attribution of iPSC approaches to future substance use or addiction research. We will not assess either the methodological quality or the risk of bias across articles. Not to mention both assessments are not required according to the scoping review methodological guidelines [104, 107, 109].

#### Stage VI. Collaborative consultation

We plan to collaborate and seek feedback on the data of our scoping review from academic and experts in stem cell research as well as substance use or addiction area. We expect their expertise and perspective will further refine the interpretation of our review results. We will request the consultants’ scientific opinion on the applications and features of the iPSC model including gaps and roadblocks along with recommendations and prospects for its applications based on our collective evidence. This information will be useful in highlighting and directing the role of iPSC approach and how to enhance the utilization of iPSC model, particularly the human iPSCs, in future substance use research.

### Ethics and dissemination

This scoping review does not involve human or living subjects; therefore, there are no need for ethical approval or informed consent. The result of our scoping review will be summarized and reported in a peer-review publication as well as an academic or scientific conference presentation.

### Limitations

The findings of the current study should be interpreted in light of several limitations. First, our review is limited by the inclusion and exclusion criteria (see the **Methods**, Stage I: ‘Inclusion and exclusion’ section). ESCs are excluded from our search strategy as our focus is on host-derived iPSCs. As such, our findings only correspond to the iPSC method, not the entirety of stem cell technologies. Second, although our review aims at wide-ranging health outcomes that are relevant to addictive substances, our findings are not reflective of patient- or treatment-oriented studies that use iPSCs to mainly examine the efficacy or response of various therapeutic agents/drugs. Third, our review is also limited to studies published in English — as such, only a fraction of the topic-related literature is summarized. Next, we will not evaluate the quality of methodologies or evidence across included papers as these are not required in scoping reviews [104, 107, 109]. Consequently, we do not intend to point out any indicators of study quality or the quality of technique protocols. Lastly, our findings will not serve as a proposed guidance or practical protocol, but rather report on the breadth of evidence and potential gaps in the application of iPSCs in the substance addiction field.

## Conclusions

A scoping review is an intuitive, systematical approach to identifying the available literature relevant to the topic of interest. We conduct a scoping review to map and present existing evidence on the application of iPSC approach in substance use/addition research, from the primary research articles published in the last 15 years. Based on the review questions, we expect our review results to provide the role and involvement of iPSC model in substance use research area.

To our knowledge, this is the first review that will provide comprehensive evidence specifically covering a broad range of basic knowledge, interplay between iPSC model and substance use research, and gaps in using iPSCs to a variety of addictive substances. Therefore, this information will advance our knowledge and help to outline future directions and challenges using iPSCs in SUD studies. As previous evidence has shown (see the **Introduction** section), the iPSC approach has proven to be invaluable in biomedical health research. Given past successes, the application of iPSC technologies to various classes of substances is likely to have a significant impact on the substance addiction field. For example, through added knowledge of etiological mechanisms and developmental process as well as greater understanding of the pharmacological and toxicological effects of drugs at cellular levels. In doing so, iPSCs may also lead to advances in the area of drug discovery/repurposing where access to living or cell-specific tissues of interest has been a significant limitation to the field [122]. In closing, it is important to note that our paper is aligned with a recent call [106] to shift the culture of conducting scoping reviews beyond clinical- or patient-oriented topics to other areas such as basic and social science studies for evidence synthesis whenever there are proper indications [98–100, 106].

## Supporting information

S1 File Preliminary PubMed® search on the topic of iPSC *AND* research areas. (PDF)

S2 File Scoping review protocol reporting checklist. (PDF)

S3 File Search terms and retrieving results for the topics of iPSC *AND* substance use disorders from the four electronic databases. (PDF)
