# Supplementary material for: On the utilization of the induced pluripotent stem cell (iPSC) model to study substance use disorders: A scoping review protocol
Source: PLoS One. 2023 Oct 12;18(10):e0292238. doi: 10.1371/journal.pone.0292238 (PMC10569547; doi:10.1371/journal.pone.0292238)
Supplement: S1 File — (PDF) [file pone.0292238.s001.pdf]

Supporting Information 1. Preliminary PubMed® search on the topic of iPSC *AND* research areas.

A

Search Strategies (Concept 1 *AND* Concept 2)		PubMed Search Details	
**Concept 1**	**Concept 2**	**Search Query**	**Records(*n*)**
iPSC	Cancers	("Induced Pluripotent"[Text Word] OR "iPS"[Text Word] OR "iPSC" [Text Word]) AND ("cancer*"[Text Word] OR "tumor*"[Text Word] OR "malignanc*"[Text Word] OR "neoplasm*"[Text Word])) AND (1991/1/1:2021/12/31[pdat])	3431
iPSC	Neurology or neuroscience or neurodegenerative disorders	(("Induced Pluripotent"[Text Word] OR "iPS"[Text Word] OR "iPSC" [Text Word]) AND ("neurology"[Text Word] OR "neuroscienc*"[Text Word] OR "neurodegenerative"[Text Word])) AND (1991/1/1:2021/12/31[pdat])	1561
iPSC	Personalized or precision medicine	(("Induced Pluripotent"[Text Word] OR "iPS"[Text Word] OR "iPSC" [Text Word]) AND ("personali*"[Text Word] OR "precision"[Text Word] OR "personalized medicine"[Text Word])) AND (1991/1/1:2021/12/31[pdat])	1086
iPSC	Psychiatric or neuropsychiatric disorders	(("Induced Pluripotent"[Text Word] OR "iPS"[Text Word] OR "iPSC" [Text Word]) AND ("psychiatr*"[Text Word] OR "neuropsychiatr*"[Text Word] OR "psychiatric disorder*"[Text Word] OR "psychiatry"[All Fields])) AND (1991/1/1:2021/12/31[pdat])	1425
iPSC	Regenerative medicine	(("Induced Pluripotent"[Text Word] OR "iPS"[Text Word] OR "iPSC" [Text Word]) AND ("regenerative medicine"[Text Word] OR "regeneration" [Text Word] OR "rejuvenation"[Text Word])) AND (1991/1/1:2021/12/31[pdat])	4257
iPSC	Substance use disorders: • Substances *AND* • Addiction *OR* dependence	(("Induced Pluripotent"[Text Word] OR "iPS"[Text Word] OR "iPSC" [Text Word]) AND ("substance*"[Text Word] OR "addict*"[Text Word] OR "abuse"[Text Word] OR "dependence"[Text Word] OR "use disorder*" [Text Word] OR "alcohol"[Text Word] OR "ethanol"[Text Word] OR "cocaine" [Text Word] OR "opioid"[Text Word] OR "smoking*"[Text Word] OR "nicotine" [Text Word] OR "cigarett*"[Text Word] OR "amphetamin*"[Text Word] OR "methamphetamin*"[Text Word] OR "marijuana"[Text Word] OR "cannabis" [Text Word])) AND (1991/1/1:2021/12/31[pdat])	733
iPSC	SUD: Alcohol/ethanol	(("Induced Pluripotent"[Text Word] OR "iPS"[Text Word] OR "iPSC" [Text Word]) AND ("substance*"[Text Word] OR "addict*"[Text Word] OR "abuse"[Text Word] OR "dependence"[Text Word] OR "use disorder*" [Text Word] OR "alcohol"[Text Word] OR "ethanol"[Text Word])) AND (1991/1/1:2021/12/31[pdat])	605
iPSC	SUD: Am-/Metham-phetamine	(("Induced Pluripotent"[Text Word] OR "iPS"[Text Word] OR "iPSC" [Text Word]) AND ("substance*"[Text Word] OR "addict*"[Text Word] OR "abuse"[Text Word] OR "dependence"[Text Word] OR "use disorder*" [Text Word] OR "amphetamin*"[Text Word] OR "methamphetamin*" [Text Word])) AND (1991/1/1:2021/12/31[pdat])	439
iPSC	SUD: Cigarette/nicotine	(("Induced Pluripotent"[Text Word] OR "iPS"[Text Word] OR "iPSC" [Text Word]) AND ("substance*"[Text Word] OR "addict*"[Text Word] OR "abuse"[Text Word] OR "dependence"[Text Word] OR "use disorder*" [Text Word] OR "smoking*"[Text Word] OR "nicotine"[Text Word] OR "cigarett*"[Text Word])) AND (1991/1/1:2021/12/31[pdat])	490
iPSC	SUD: Cannabis	("Induced Pluripotent"[Text Word] OR "iPS"[Text Word] OR "iPSC" [Text Word]) AND ("substance*"[Text Word] OR "addict*"[Text Word] OR "abuse"[Text Word] OR "dependence"[Text Word] OR "use disorder*" [Text Word] OR "marijuana"[Text Word] OR "cannabis"[Text Word])) AND (1991/1/1:2021/12/31[pdat])	435
iPSC	SUD: Cocaine	(("Induced Pluripotent"[Text Word] OR "iPS"[Text Word] OR "iPSC" [Text Word]) AND ("substance*"[Text Word] OR "addict*"[Text Word] OR "abuse"[Text Word] OR "dependence"[Text Word] OR "use disorder*" [Text Word] OR "cocaine"[Text Word])) AND (1991/1/1:2021/12/31[pdat])	432
iPSC	SUD: Opioid	(("Induced Pluripotent"[Text Word] OR "iPS"[Text Word] OR "iPSC" [Text Word]) AND ("substance*"[Text Word] OR "addict*"[Text Word] OR "abuse"[Text Word] OR "dependence"[Text Word] OR "use disorder*" [Text Word] OR "opioid"[Text Word])) AND (1991/1/1:2021/12/31[pdat])	471

B-1

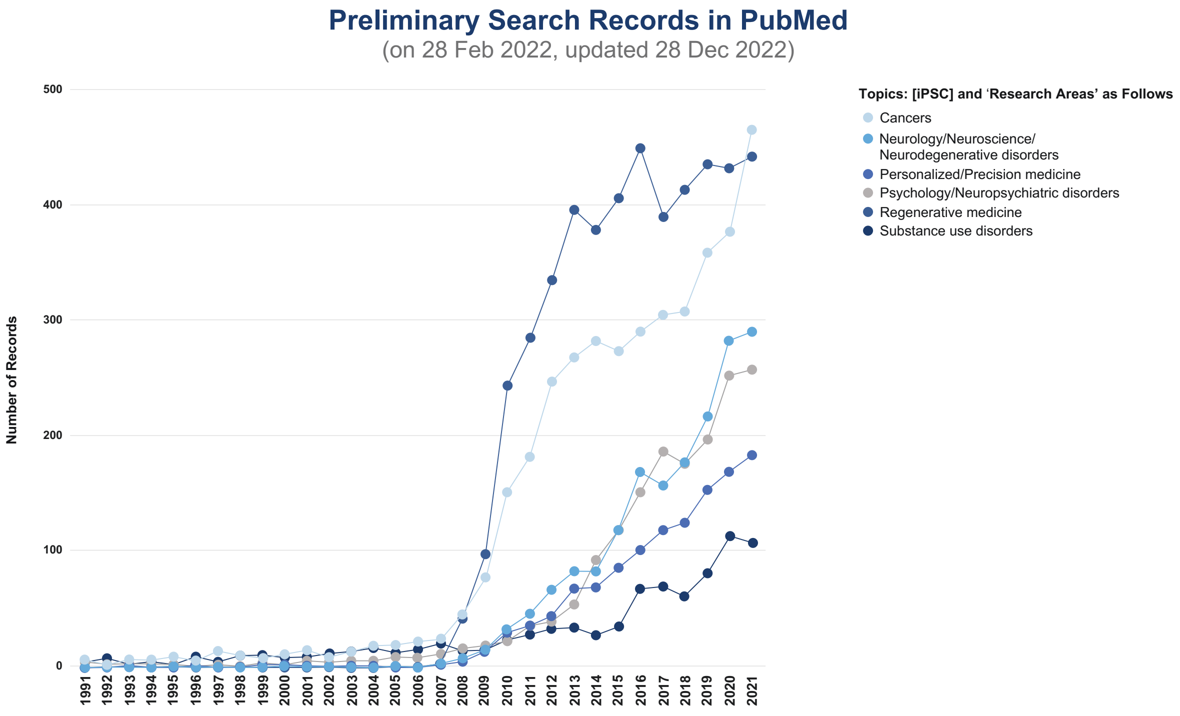
Preliminary Search Records in PubMed
(on 28 Feb 2022, updated 28 Dec 2022)
Number of Records
Topics: [iPSC] and 'Research Areas' as Follows
Cancers
Neurology/Neuroscience/ Neurodegenerative disorders
Personalized/Precision medicine
Psychology/Neuropsychiatric disorders
Regenerative medicine
Substance use disorders

B-2

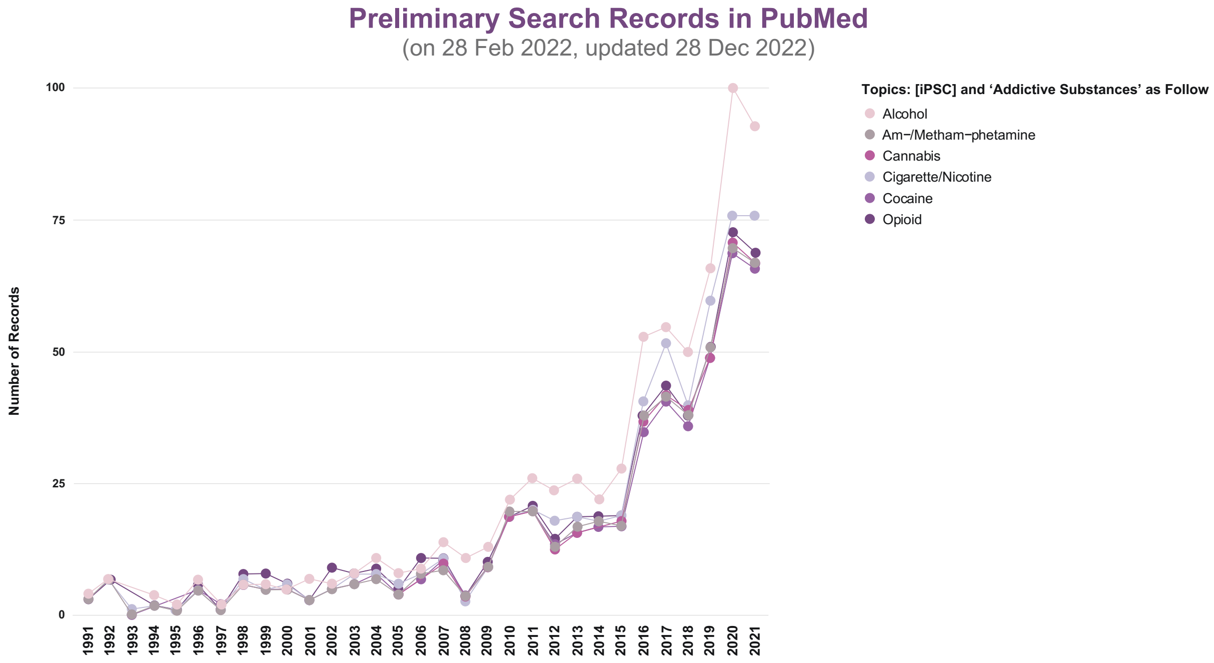
Preliminary Search Records in PubMed
(on 28 Feb 2022, updated 28 Dec 2022)
Number of Records
Topics: [iPSC] and 'Addictive Substances' as Follow
Alcohol
Am−/Metham−phetamine
Cannabis
Cigarette/Nicotine
Cocaine
Opioid

Supporting Information 1 summarizes the preliminary search on the topic of **iPSC *AND* research areas** from **PubMed®** (*The United States National Library of Medicine, the National Institutes of Health,* Bethesda, MD, USA) database starting from 1991 to 2021. We examined a total of 12 research areas, comprised of six notable fields (cancers, neurology/neuroscience/-neurodegenerative disorders, personalized/precision medicine, psychiatric/neuro-psychiatric disorders, regenerative medicine, substance use disorders) and six specific common substance use disorders (alcohol, am-/metham-phetamine, cigarette/nicotine, cannabis, cocaine, and opioid). The search was originally retrieved on 22 February 2022 and updated on 28 December 2022.

Overall, the search results showed that the records of iPSC in each research area started growing after the first human iPSC reprogramming was generated in 2007 [1], and then has become accelerating in various research fields after the clinical iPSC trial was launched in 2014 [2, 3]. In our search results, among the six major research areas, the top field was regenerative medicine, followed by cancers, while the least prevalent was substance use disorders. Moreover, among the six common substances used, the highest number of records was alcohol, followed by cigarette/nicotine. The number of records for substance use disorders as well as alcohol and cigarette/nicotine uses were far smaller than the other five research areas. Altogether, this information suggests a plausible conclusion that the iPSC model has been less used in substance use areas than other major research areas, including other psychiatric disorders. Noticeably, among the six common substances used, alcohol and cigarette/nicotine have been the most frequently studied using iPSC approaches.

1A. Summary table for the search terms and results.

As this is a preliminary assessment to determine a prospective amount of available literature in our topic of interest, we did a PubMed search using gross search terms as shown in this tableau **Supporting Information 1A**. **Two concepts** were applied as follows: (**i**) **Concept 1** – 'iPSC' (*the 1st column*) ***AND*** (**ii**) **Concept 2** – 'research areas' (*the 2nd column*) with the limited year of publication from 1991 to 2021. Each of the search terms and results for the 12 research areas is presented in each row and grouped by colored according to relevance to a specific substance use disorder: *irrelevance* (rows 1-6, blue bars) and *relevance* (rows 7-12, purple bars). The search terms and results for each research area are detailed in *the 3rd and 4th columns*, respectively. The search results are presented as the horizontal bars with numbers of search records (*the 4th column*).

1B. Scatter plots of the search results.

Scatter plots show the PubMed search results focused on **iPSC *AND* research areas** during the publication time period from 1991 to 2021. The graph plots the data by the year of publication (x-axis) against the number of search records (y-axis) published in that

year. The line between points displays a trend of records over time (year range: 1991-2021; 31 years). Colors indicate specific research areas.

- **1B-1.** The plot represents the PubMed search results for the topic of **iPSC *AND* the six main research areas** as follows:
  - Cancers
  - Neurology/neuroscience/neurodegenerative disorders
  - Personalized/precision medicine
  - Psychiatric/neuropsychiatric disorders
  - Regenerative medicine
  - Substance use disorders

- **1B-2.** The plot represents the PubMed search results for the topic of **iPSC *AND* the six specific substance use disorders** as follows:
  - Alcohol
  - Am-/Metham-phetamine
  - Cannabis
  - Cigarette/nicotine
  - Cocaine
  - Opioid

iPSC, induced pluripotent stem cell; SUD, substance use disorder.

References:

1. Takahashi K, Tanabe K, Ohnuki M, Narita M, Ichisaka T, Tomoda K, et al. Induction of pluripotent stem cells from adult human fibroblasts by defined factors. Cell. 2007;131(5):861-72. doi:10.1016/j.cell.2007.11.019.
2. Jin ZB, Okamoto S, Osakada F, Homma K, Assawachananont J, Hirami Y, et al. Modeling retinal degeneration using patient-specific induced pluripotent stem cells. PLoS One. 2011;6(2):e17084. doi:10.1371/journal.pone.0017084.
3. Mandai M, Watanabe A, Kurimoto Y, Hirami Y, Morinaga C, Daimon T, et al. Autologous Induced stem-cell-derived retinal cells for macular degeneration. N Engl J Med. 2017;376(11):1038-46. doi:10.1056/NEJMoa1608368.
