# Supplementary material for: On the utilization of the induced pluripotent stem cell (iPSC) model to study substance use disorders: A scoping review protocol
Source: PLoS One. 2023 Oct 12;18(10):e0292238. doi: 10.1371/journal.pone.0292238 (PMC10569547; doi:10.1371/journal.pone.0292238)
Supplement: S2 File — (PDF) [file pone.0292238.s002.pdf]

Supporting Information 2. Scoping review protocol reporting checklist.

SCOPING REVIEW GUIDELINE OF REPORTING PROTOCOL

Section	Topic		Item	Checklist Item[a]	Section Found: STUDY PROTOCOL
**ADMINISTRATIVE INFORMATION**				
Title	Identification	1a	Identify the report as a protocol	Title
	Update protocol	1b	identify an update, if a previous protocol exists	NA
Registration		2	If registered, provide the name of the registry (such as JBI) and registration number	Methods: Study protocol
Authors	Contact	3a	Provide name, institutional affiliation, e-mail address of all protocol authors; provide physical mailing address of corresponding author	Co-authors' info; Corresponding author's info
	Contributions	3b	Describe contributions of protocol authors and identify the guarantor of the review	Author Contributions
Amendments		4	If the protocol represents an amendment of a previously completed or published protocol, identify as such and list changes; otherwise, state plan for documenting important protocol amendments	Methods: Study protocol
Support	Sources	5a	Indicate sources of financial or other support for the review	Funding
	Sponsor	5b	Provide name for the review funder and/or sponsor	Funding
	Role of sponsor or funder	5c	Describe roles of funder(s), sponsor(s), and/or institution(s), if any, in developing the protocol	Disclaimer
**INTRODUCTION**				
Rationale		6	Describe the rationale for the review in the context of what is already known (Note: Consider providing a rationale for the choice of conducting a scoping review as compared to other evidence synthesis approaches)	Study rationale
Objectives		7	Provide an explicit statement of the question(s) the review will address with reference to the inclusion/ exclusion criteria	Methods: Stage I, Review objectives/questions, para 1-3
**METHODS**				
Eligibility criteria		8	Specify the study characteristics (such as PICO, study design, setting, time frame) and report characteristics (such as years considered, language, publication status) to be used as criteria for eligibility for the review	Methods: Stage I, Inclusion and exclusion criteria
Information sources		9	Describe all intended information sources (such as electronic databases, contact with study authors, trial registers or other grey literature sources) with planned dates of coverage	Methods: Stage II, Sources and types of evidence
Search strategy		10	Present draft of search strategy to be used for at least one electronic database, including planned limits, such that it could be repeated	Methods: Stage II, Search strategy and terms
Study records	Data management	11a	Describe the mechanism(s) that will be used to manage records and data throughout the review	Methods: Stage III, Study selection, para 1-3
	Selection process	11b	State the process that will be used for selecting studies (such as two independent reviewers) through each phase of the review (that is, screening, eligibility and inclusion)	Methods: Stage III, Study selection, para 3-5
	Data collection process	11c	Describe planned method of extracting data from reports (such as piloting forms, done independently, in duplicate), any processes for obtaining and confirming data from investigators	Methods: Stage IV, Data extraction, para 1, 2
Data items		12	List and define all variables for which data will be sought (such as PICO items, funding sources), any preplanned data assumptions and simplifications (Note: Scoping reviews may not use PICO and instead may use JBI's Population, Concept, and Context [PCC] or another approach to reporting eligibility criteria)	Methods: Stage IV, Data extraction, para 1, 3
Outcomes and prioritization		13	List and define all outcomes for which data will be sought, including prioritization of main and additional outcomes, with rationale (Note: Scoping reviews may not extract outcome data, so this can refer to whichever data items are extracted)	Methods: Stage IV, Data extraction, para 3
Risk of bias in individual studies		14	If this is to occur, describe anticipated methods for assessing risk of bias of individual studies, including whether this will be done at the outcome or study level, or both; state how this information will be used in data synthesis (Note: Scoping reviews typically do not include risk of bias assessment, but this information should be described if it will occur)	NA

Section | Topic		Item	Checklist Item[a]	Sections Found: STUDY PROTOCOL
**Data**				
Data synthesis		15a	Describe criteria under which study data will be presented (Note: Scoping reviews do not typically include quantitative synthesis of study data, but should still describe in advance how extracted data are anticipated to be presented in the resulting review)	Methods: Stage V, Data analysis and presentation, para 1
		15b	Describe the planned approach to how extracted data will be presented (such as figures, tables, evidence gaps maps)	
		15c	Describe any proposed additional analyses (such as thematic analyses) (Note: The JBI methodological guidance does not recommend undertaking thematic analysis as this synthesis of data should ideally occur following methodological appraisal of the included sources)	NA
		15d	If quantitative synthesis is not appropriate, describe the type of summary planned	Methods: Stage V, Data analysis and presentation, para 2
Meta-bias(es)		16	Specify any planned assessment of meta-bias(es) (such as publication bias across studies, selective reporting within studies) (Note: Scoping reviews typically do not include assessment of meta-bias[es], but this information should be described if it will occur)	NA
Confidence in cumulative evidence		17	Describe how the strength of the body of evidence will be assessed (such as GRADE)	NA

*** It is strongly recommended that this checklist be read in conjunction with the PRISMA-P Explanation and Elaboration by Shamseer et al.[5] for important clarification on the items. Amendments to a review protocol should be tracked and dated. The copyright for PRISMA-P (including checklist) is held by the PRISMA-P Group and is distributed under a Creative Commons Attribution Licence 4.0.**

GRADE, Grading of Recommendations, Assessment, Development and Evaluation.
NA, Not applicable.
para, paragraph.

Adapted from: Shamseer L, Moher D, Clarke M, Ghersi D, Liberati A, Petticrew M, et al. PRISMA-P Group. Preferred reporting items for systematic review and meta-analysis protocols (PRISMA-P) 2015: elaboration and explanation. BMJ. 2015;349:g7647. Table 2, PRISMA-P (preferred reporting items for systematic review and meta-analysis protocols) 2015 checklist: recommended items to address in a systematic review protocol; p.24. CC BY 4.0.

[a]Data of checklist *with permission* from: Peters MDJ, Godfrey C, McInerney P, Munn Z, Tricco AC, Khalil H. Best practice guidance and reporting items for the development of scoping review protocols. JBI Evid Synth. 2022;20(4):953-68. Appendix I, Recommended items to address in a scoping review protocol; p.967-8.

Supporting Information 2 illustrates a reporting checklist for scoping review protocols. The Preferred Reporting Items for Systematic Reviews and Meta-Analyses (PRISMA-P) checklist [1, 2] was developed to help preparing and reporting of systematic review protocol and also adapted to scoping review protocol. The PRISMA-P 2015 checklist includes 17 main items [1] of which some may not be compelled for scoping reviews due to the differences in objectives and methodologies between systematic reviews and scoping reviews. Recently, the PRISMA-P for scoping review protocol was further augmented by the Joanne Briggs Institute (JBI) Scoping Review Methodology Group published in 2022 [3].

Here, we reported the recommended items in the protocol based on the guidelines from the PRISMA-P 2015 [1, 2] and the JBI 2022 [3]. Checklist items that can be found in our scoping review protocol are listed in the last column, '**Section found**'.

NA, not applicable; para, paragraph.

References:

1. Moher D, Shamseer L, Clarke M, Ghersi D, Liberati A, Petticrew M, et al. Preferred reporting items for systematic review and meta-analysis protocols (PRISMA-P) 2015 statement. Syst Rev. 2015;4(1):1. doi:10.1186/2046-4053-4-1.
2. Shamseer L, Moher D, Clarke M, Ghersi D, Liberati A, Petticrew M, et al. Preferred reporting items for systematic review and meta-analysis protocols (PRISMA-P) 2015: elaboration and explanation. BMJ. 2015;350:g7647. doi:10.1136/bmj.g7647.
3. Peters MDJ, Godfrey C, McInerney P, Khalil H, Larsen P, Marnie C, et al. Best practice guidance and reporting items for the development of scoping review protocols. JBI Evid Synth. 2022;20(4):953-68. doi:10.11124/JBIES-21-00242.
