# Supplementary material for: On the utilization of the induced pluripotent stem cell (iPSC) model to study substance use disorders: A scoping review protocol
Source: PLoS One. 2023 Oct 12;18(10):e0292238. doi: 10.1371/journal.pone.0292238 (PMC10569547; doi:10.1371/journal.pone.0292238)
Supplement: S3 File — (PDF) [file pone.0292238.s003.pdf]

Supporting Information 3. Search terms and retrieving results for the topics of iPSC ***AND*** substance use disorders from the four electronic databases.

A

PubMed.gov

History and Search Details (Searched: 15 April 2022)

Search	Actions	Details	Query	Results
#3	•••	>	Search: **(#1 AND #2) AND English[lang] AND "Journal Article" [Publication Type] AND ("2007/01/01"[Date - Publication] : "2022/03/31"[Date - Publication])**	1,327
#2	•••	>	Search: **(Substance*[tw] OR Alcohol*[tw] OR ethanol*[tw] OR Narcotic*[tw] OR Cocaine[tw] OR Opioid*[tw] OR amphetamine*[tw] OR meth[tw] OR methamphetamine*[tw] OR marijuana[tw] OR cannab*[tw] OR nicotin*[tw] OR tobacco[tw] OR cigar*[tw] OR Smoke*[tw] OR Smoking[tw] OR Psychoactive[tw] OR psychostimulant*[tw] OR MPTP[tw] OR MDA[tw] OR MDMA[tw] OR Addict*[tw] OR Abus*[tw] OR Habit*[tw] OR Misuse*[tw] OR User*[tw] OR hallucinoge*[tw] OR "illicit drug*"[tw] OR "illegal drug*"[tw] OR depressants[tw])**	2,288,622
#1	•••	>	Search: **("Induced Pluripotent"[tw] OR IPS[tw] OR IPSC[tw] OR hiPSC[tw] OR organoid*[tw] OR "pluripotent stem cell*"[tw])**	59,063

B

Embase

(Searched: 4 April 2022)

History Save | Delete | Print view | Export | Email Combine > using And Or ^ Collapse

#4	#3 AND 'article'/it	961
#3	#1 AND #2 AND [English]/lim AND [01-01-2007]/sd NOT [01-04-2022]/sd	1,919
#2	**substance***:ti,ab,kw OR **alcohol***:ti,ab,kw OR **ethanol***:ti,ab,kw OR **narcotic***:ti,ab,kw OR **cocaine**:ti,ab,kw OR **opioid***:ti,ab,kw OR **amphetamine***:ti,ab,kw OR **meth**:ti,ab,kw OR **methamphetamine***:ti,ab,kw OR **marijuana**:ti,ab,kw OR **cannab***:ti,ab,kw OR **nicotin***:ti,ab,kw OR **tobacco**:ti,ab,kw OR **cigar***:ti,ab,kw OR **smoke***:ti,ab,kw OR **smoking**:ti,ab,kw OR **psychoactive**:ti,ab,kw OR **psychostimulant***:ti,ab,kw OR **mptp**:ti,ab,kw OR **mda**:ti,ab,kw OR **mdma**:ti,ab,kw OR **addict***:ti,ab,kw OR **abus***:ti,ab,kw OR **habit***:ti,ab,kw OR **misuse***:ti,ab,kw OR **user***:ti,ab,kw OR **hallucinoge***:ti,ab,kw OR "**illicit drug***":ti,ab,kw OR "**illegal drug***":ti,ab,kw OR **depressants**:ti,ab,kw	2,487,029
#1	"**induced pluripotent**":ti,ab,kw OR **ips**:ti,ab,kw OR **ipsc**:ti,ab,kw OR **hipsc**:ti,ab,kw OR **organoid***:ti,ab,kw OR "**pluripotent stem cell***":ti,ab,kw	63,151

C

Clarivate

Web of Science™

(Searched: 4 April 2022)

0/3 Combined Sets Export Clear History

3	(((#2 AND #1) AND DT=(Article)) AND PY=(2007-2022)) AND LA=(English)	1,438	Add to Query
2	TS=(Substance* OR Alcohol* OR ethanol* OR Narcotic* OR Cocaine OR Opioid* OR amphetamine* OR meth OR methamphetamine* OR marijuana OR cannab* OR nicotin* OR tobacco OR cigar* OR Smoke* OR Smoking OR Psychoactive OR psychostimulant* OR MPTP OR MDA OR MDMA OR Addict* OR Abus* OR Habit* OR Misuse* OR User* OR hallucinoge*OR "illicit drug*" OR "illegal drug*" OR depressants)	3,733,143	Add to Query
1	TS=("Induced Pluripotent" OR IPS OR IPSC OR hiPSC OR organoid* OR "pluripotent stem cell*")	62,668	Add to Query

D

Scopus

Search History Saved Searches

(Searched: 1 April 2022)

Combined queries

2	TITLE-ABS-KEY (("induced Pluripotent" OR ips OR ipsc OR hipsc OR organoid* OR "pluripotent stem cell*")) AND (TITLE-ABS-KEY (substance* OR alcohol* OR ethanol* OR narcotic* OR cocaine OR opioid* OR amphetamine* OR meth OR methamphetamine* OR marijuana OR cannab* OR nicotin* OR tobacco OR cigar* OR smoke* OR smoking OR psychoactive OR psychostimulant* OR mptp OR mda OR mdma OR addict* OR abus* OR habit* OR misuse* OR user* OR hallucinoge* OR "illicit drug*" OR "illegal drug*" OR depressants)) AND PUBYEAR > 2006 AND (LIMIT-TO (DOCTYPE , "ar") OR LIMIT-TO (DOCTYPE , "English")) Show less	2,257 results Set Alert More
1	TITLE-ABS-KEY (("induced Pluripotent" OR ips OR ipsc OR hipsc OR organoid* OR "pluripotent stem cell*")) AND (TITLE-ABS-KEY (substance* OR alcohol* OR ethanol* OR narcotic* OR cocaine OR opioid* OR amphetamine* OR meth OR methamphetamine* OR marijuana OR cannab* OR nicotin* OR tobacco OR cigar* OR smoke* OR smoking OR psychoactive OR psychostimulant* OR mptp OR mda OR mdma OR addict* OR abus* OR habit* OR misuse* OR user* OR hallucinoge* OR "illicit drug*" OR "illegal drug*" OR depressants)) AND PUBYEAR > 2006 Show less	3,426 results Set Alert More

Supporting Information 3 displays search terms and retrieving results for the topics of **induced pluripotent stem cell** (iPSC) ***AND*** **substance use disorders** from four electronic databases: **PubMed**® (*The United States National Library of Medicine, the National Institutes of Health,* Bethesda, MD, USA), **Embase**® (*Elsevier,* Amsterdam, Netherlands), **Web of Science**™ Core Collection (*Science Citation Index Expanded, Social Sciences Citation Index, Arts & Humanities Citation Index, Emerging Sources Citation Index, Conference Proceedings Citation Index, Book Citation Index, and Current Chemical Reactions, Index Chemicus; Clarivate*™, London, UK), and **Scopus**® (*Elsevier,* Amsterdam, Netherlands).

Two concepts were applied: (**i**) **Concept 1 – iPSC:** ("induced pluripotent" OR iPS OR iPSC OR hiPSC OR organoid* OR "pluripotent stem cell*") ***AND*** (**ii**) **Concept 2 – addictive drugs or substances:** (substance* OR alcohol* OR ethanol* OR narcotic* OR cocaine OR opioid* OR amphetamine* OR meth OR methamphetamine* OR marijuana OR cannab* OR nicotin* OR tobacco OR cigar* OR smoke* OR smoking OR psychoactive OR psychostimulant* OR MPTP OR MDA OR MDMA OR addict* OR abus* OR habit* OR misuse* OR user* OR hallucinoge* OR "illicit drug*" OR "illegal drug*" OR depressants). The search was limited the publication time from 2007 to March 2022. Details about search strategy, including concepts can be found in the protocol, the **Methods**, Stage II: 'Search strategy and terms' section and **Fig 4A**.

The search queries and indexed terms were adapted and applied to the search engine of each database. Search terms and results for each database are shown as follows:

- **3A.** PubMed® search and results.
- **3B.** Embase® search and results.
- **3C.** Web of Science™ search and results.
- **3D.** Scopus® search and results.
